# Combatting over-specialization bias in growing chemical databases

**DOI:** 10.1186/s13321-023-00716-w

**Published:** 2023-05-19

**Authors:** Katharina Dost, Zac Pullar-Strecker, Liam Brydon, Kunyang Zhang, Jasmin Hafner, Patricia J. Riddle, Jörg S. Wicker

**Affiliations:** 1 School of Computer Science, University of Auckland, 38 Princes Street, 1010 Auckland, New Zealand; 2 enviPath UG & Co. KG, In den Graswiesen 13, 55437 Ockenheim, Germany; 3 Eawag-Swiss Federal Institute of Aquatic Science and Technology, Überlandstrasse 133, 8600 Dübendorf, Switzerland

**Keywords:** Machine learning, Bias, Data quality, Chemical compound space

## Abstract

**Background:**

Predicting in advance the behavior of new chemical compounds can support the design process of new products by directing the research toward the most promising candidates and ruling out others. Such predictive models can be data-driven using Machine Learning or based on researchers’ experience and depend on the collection of past results. In either case: models (or researchers) can only make reliable assumptions about compounds that are similar to what they have seen before. Therefore, consequent usage of these predictive models shapes the dataset and causes a continuous specialization shrinking the applicability domain of all trained models on this dataset in the future, and increasingly harming model-based exploration of the space.

**Proposed solution:**

In this paper, we propose cancels (**C**ounter**A**cti**N**g **C**ompound sp**E**cia**L**ization bia**S**), a technique that helps to break the dataset specialization spiral. Aiming for a smooth distribution of the compounds in the dataset, we identify areas in the space that fall short and suggest additional experiments that help bridge the gap. Thereby, we generally improve the dataset quality in an entirely unsupervised manner and create awareness of potential flaws in the data. cancels does not aim to cover the entire compound space and hence retains a desirable degree of specialization to a specified research domain.

**Results:**

An extensive set of experiments on the use-case of biodegradation pathway prediction not only reveals that the bias spiral can indeed be observed but also that cancels produces meaningful results. Additionally, we demonstrate that mitigating the observed bias is crucial as it cannot only intervene with the continuous specialization process, but also significantly improves a predictor’s performance while reducing the number of required experiments. Overall, we believe that cancels can support researchers in their experimentation process to not only better understand their data and potential flaws, but also to grow the dataset in a sustainable way. All code is available under github.com/KatDost/Cancels.

## Introduction

In domains where gathering data requires time-intensive experiments, predicting likely outcomes for experiments helps concentrate efforts on the right experiments. One example is the development of effective yet sustainable and environmentally-friendly products, e.g., pesticides, that (hopefully) fulfill their purpose and then quickly degrade into harmless non-toxic compounds over time. Experiments involve long-term studies of each compound’s effect and observation in soil under different environmental conditions. Ruling out compounds that might not bring the desired chemical properties or degrade into toxic by-products is an essential aspect of the development process. Similar challenges arise in other areas of chemical research and development, such as the design of new pharmaceuticals, fragrances, or commodity chemicals.

However, predictive models learn from and specialize to the data provided to them [1, 2]. While this specialization is useful up to the point where the desired domain is accurately captured [3, 4], the models can over-specialize. Starting from the initial dataset, a trained model will only be able to make reliable predictions in densely populated areas of the compound space, leaving the remaining areas outside of the model’s applicability domain. As a consequence, it will suggest a set of experiments well within its applicability domain, shifting the overall data distribution towards in-domain data. Should the model be re-trained after obtaining the new experimental results, it will put more emphasis on the now densely populated areas further shifting the data distribution. After a few iterations of dataset growth, we can observe that the applicability domain is either consistent or shrinking despite the additional data [5], and new potentially interesting areas of the compound space will never be explored. For example, in density-based applicability domain techniques using relative thresholds [6, 7], the density ratio between dense and sparse areas changes—and rightfully so since a trained model will increasingly focus on dense areas and become less reliable on sparse ones. This scenario is a self-reinforcing type of selection bias where the model chooses to obtain new results for compounds it can already predict reliably, and therefore slows down or even stops learning.

A similar effect can be observed when humans rather than models choose the compounds to experiment with [8]. Jia et al. [9] argue that anthropogenic factors play a key role in the compound selection process for experiments, and hence the development of datasets. More than on the cost, availability, or ease of use of available candidate compounds, researchers tend to base their selection on their past successes and that of their colleagues or research articles. This results in a specialization spiral iteratively narrowing down the scope within which models and humans can make informed decisions.

Active Learning [10] is a tool that aims to break the cycle by selecting the most informative experiments for the model instead. Although Active Learning has been shown to suffer from shifts in distribution [11], it is capable of slowly expanding the compound space and will eventually even explore beyond the desired degree of specialization. In addition, Active Learning is always model-dependent. This is a major drawback since datasets, especially those requiring long-term experiments, can and will be used for different purposes over time, and it is often infeasible to gather new data specifically for a model.

Instead, in this paper, we suggest cancels (**C**ounter**A**cti**N**g **C**ompound sp**E**cia**L**ization bia**S**), a model-free and even task-free method to generally point out potential shortcomings of the data and improve the quality without losing the desired specialization to a specific domain.

In the Machine Learning landscape, two algorithms have been proposed that are specifically designed to search for dataset issues induced by the sampling process without requiring additional information such as a ground-truth sample or distribution. imitate [12] and mimic [13] investigate the dataset’s distribution and hint to flaws that might be a consequence of a selection *bias*, that is, a mismatch between the probability distributions of a non-uniformly drawn sample and the sampling space. Although these mismatches are not always visible from the biased sample alone, both methods identify unusual and sharp deviations in density that will cause issues for modeling tasks, and they generate additional data points to smooth out the distribution. Both methods operate under different assumptions regarding the definition of flaws but are designed for real-valued tabular data which is not provided by chemical compound datasets. Additionally, the methods’ generation of artificial compounds that mitigate the bias could result in infeasible compounds that are neither useful nor interpretable. While the general idea of imitate and mimic aligns with the problem we attempt to solve in this paper, neither is directly applicable.

cancels adapts ideas from both and extends them to select data from a pre-defined pool rather than generating which allows us the freedom to select meaningful compounds worth experimenting with from a data quality standpoint. Possible applications for cancels include Computer-Aided Drug Design (CADD) [14, 15]. These methods greatly support the drug discovery and development process by modeling the behavior of compounds, but, as is common in all data-based methods such as Machine Learning, they can only make reliable predictions for compounds that are similar to what those models trained on [16]. This might be one of the key reasons why, despite the progress of CADD methods in recent years, still only a small fraction of the chemical compound space has been explored in the search for drug candidates (as stated by Mouchlis et al. [14]). While *de novo* drug design [2, 16–18] aims to base the candidate search on a broader space, it also relies on the quality of the underlying dataset [19, 20], and it disregards the distributions of the resulting compound set and their implications for future predictors or generators [4]. cancels can help select additional compounds to test in order to improve the dataset quality for future drug design cycles while still testing the most promising candidates for today’s search.

The remainder of this section discusses the problem we attempt to solve and reviews related research. The "Proposed method" section introduces the cancels algorithm. Based on the experimental setup outlined in the "Experimental setup" section, the "Results and discussion" section presents and discusses experimental results. Finally, we conclude the paper.

**Fig. 1:**
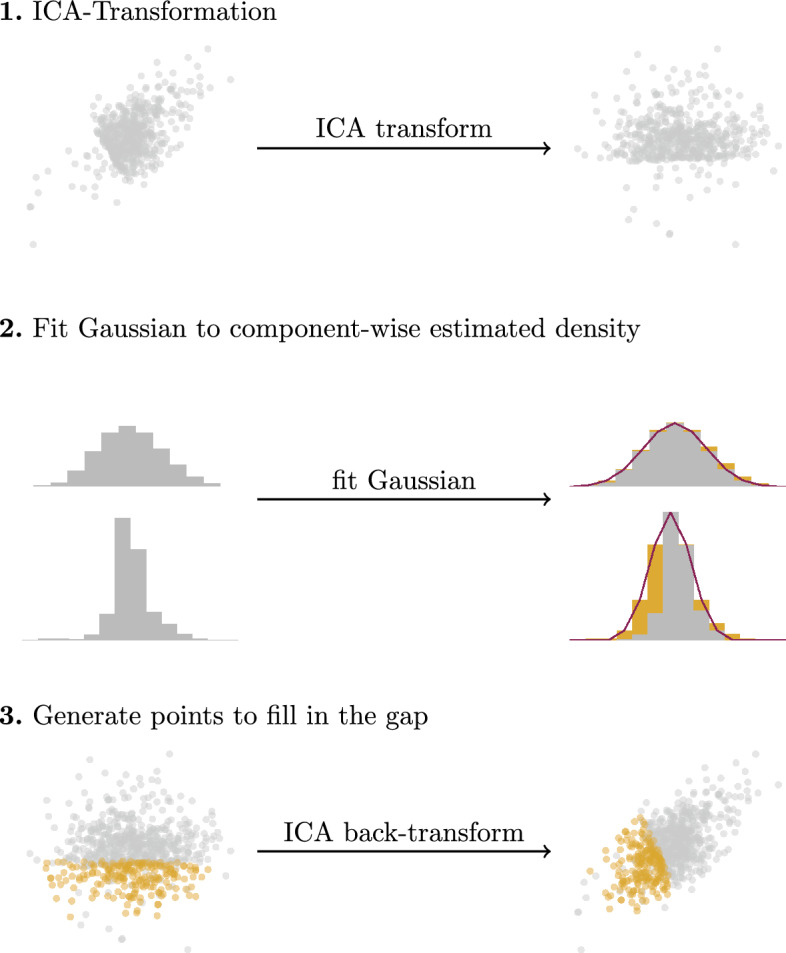
Overview over the imitate algorithm

### Background

Aiming to support the data gathering process and improve the data quality on-the-fly, an understanding of flaws and shortcomings in the dataset is crucial as it allows us to smooth them out with subsequent experiments. Typically, no perfect and complete sample is available that could be used as a ground truth to compare with and strive for as it would render the gathering process obsolete. While missing values and sparsely populated areas of the compound space are simple to detect, biases are often less visible. Yet they compromise the training of models and inferred conclusions limiting the scope and precision of future discoveries [1, 2]. Therefore, in this paper, we aim to create awareness of potential biases and grant the researcher the opportunity to mitigate their effects early on, independently of the models that can arise from the collected data. Formally, we state the problem we aim to solve as follows:

**Problem statement.** *Let* $D$ *be an (unknown) compound dataset (potentially with labels or properties) that is representative of an underlying distribution which we consider to be the ground truth. Given only a biased subset* $B\subset D$ *and a pool* $P$ *of candidate compounds, the task is to select a set of compounds* $P_\text {sel} \subseteq P$ *such that a model trained on* $B\cup P_\text {sel}$ *would provide minimally different outputs (such as predictions, clusters, etc.) from one trained on* $D$.

The problem is adapted from the reconstruction problem we first introduced in 2020 [12], where, instead of selecting from a pool, we generated additional data points. We presented the imitate algorithm that, given only the biased dataset *B*, generates additional data to mitigate the bias. While imitate is limited to normally distributed data, mimic [13] extends its scope to datasets that can be modeled as mixtures of Gaussians. The assumption of normality is well motivated for three reasons: First, intuitively, we would expect a trained model to perform well on the domain it is designed for, and we allow a certain amount of error around the fringes and would not expect it to perform on entirely different data. This describes a Gaussian-like distribution of the underlying dataset. We also expect a reasonably smooth data distribution, particularly for larger datasets. Second, Bareinboim et al. [21] prove theoretically that, without additional data or assumptions, the true class label distributions cannot be recovered from the biased data. Hence, trained models will not generalize well. Therefore, some assumption is necessary. Third, normal distributions are very common in nature [22] as a consequence of the Central Limit Theorem.^1^

However, we can assume that not all distributions we might encounter are normally distributed. To avoid misleading results in this case, both methods test if a Gaussian fits the data reasonably well and refuse any further outputs if not. See the original papers for details on the definition of a ‘reasonable’ fit in this case. Since the observed dataset is potentially biased skewing its distribution, the acceptable margin necessarily needs to be sufficiently large. Hence, if the true data distribution is similar to (but not exactly) a Gaussian, this distinction will likely not be detected. But since we can expect smoothing over the data distribution to improve the data quality regardless, the implications of assuming a Gaussian distribution are overall benevolent.

Because we use parts of both imitate and mimic for our research, we present them here briefly and refer the interested reader to the original papers for more details.

**Fig. 2:**
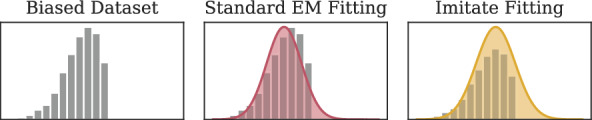
Comparison of the Gaussians fitted to a biased dataset (left) when using the traditional Expectation-Maximization fitting (center) and the fitting procedure outlined in the imitate algorithm (right)

As illustrated in Fig. 1, given only a biased dataset *B*, imitate [12] uses *Independent Component Analysis (ICA)* [24] to transform it into a new space. There, the axes are statistically independent and chosen in a way that they show those data distributions that resemble a Gaussian the least. Keeping imitate’s assumption that the dataset’s ground truth follows a normal distribution in mind, this transformation exposes *B*’s weaknesses and allows for component-wise analysis of the data. After transforming the data to the new space found with ICA, imitate analyzes the data for each of the axes separately. It represents the data density with a histogram or grid-like evaluation of a kernel density estimator and heuristically aims to find the Gaussian that most likely represents the ground-truth distribution under a selection bias scenario. To find a good fit, imitate uses the bin positions and heights to fit a Gaussian density function using an ordinary least squares optimizer. To put more emphasis on the observed data than the data that is potentially missing due to a bias, imitate adjusts the weights during the optimization to the bin heights. Note that this procedure yields fundamentally different results from traditional density fitting techniques using the Expectation-Maximization approach if a selection bias is present since instead of modeling the present data *B*, imitate aims to capture the potential ground truth *D*. See Fig. 2 for a comparison. Once the Gaussians have been fitted for all components, imitate generates points to fill in the gap between the Gaussian and the observed density and back-transforms them to the original data space. If these generated points focus on certain areas, they indicate a potential selection bias. Suitable visualization of these areas can help the researcher understand the dataset and its potential flaws. Additionally, if a bias has been identified, adding the generated data points to the biased dataset *B* before training a model can help improve its performance.

Because imitate fits only one Gaussian per dimension to the data, it is limited to datasets whose ground truth can be expected to contain only one normally distributed cluster. mimic relaxes this limitation and divides the data into presumably Gaussian clusters before applying the imitate algorithm to each of them separately. The key element of mimic is the clustering itself. As opposed to typical clustering approaches that find the optimal separation of the present data, mimic aims to cluster the ground truth instead, given only the biased data. Starting from small non-Gaussian clusters obtained via, for example, KMeans, mimic iteratively applies imitate to the cluster to identify where points need to be added to obtain a smooth Gaussian. When possible, mimic selects those points from other clusters that fill in the gap best, re-applies imitate, assigns further points, etc. until either a smooth Gaussian is achieved or no suitable points are available. Finally, mimic resolves overlapping clusters by merging and uses imitate for each of these clusters to generate and analyze points that can indicate and mitigate a bias.

Both methods have been shown to help mitigate selection biases on real-valued tabular data [12, 13] but have not yet been successfully applied to special types of data incorporating, for example, categorical or even binary features. Additionally, they cannot account for natural limitations or boundaries of the data space, and random generation of data points in the chemical compound space will most likely result in impossible compounds misleading models rather than helping them. Aiming to gain an understanding of chemical compound datasets and their inherent biases, in this paper, we present ways to utilize (parts of) both methods in our context and overcome the issues mentioned above.

### Related work

Except for imitate and mimic, to the best of our knowledge, no method has been proposed to investigate biases without using a ground-truth sample or additional information about the bias. In this section, we review fields that deal with related problems, i.e., bias detection with ground-truth samples and active learning for chemistry, and highlight the differences to our problem statement. Additionally, we discuss biases specifically in the chemical compound space.

*Bias detection using ground-truth information*

The goal of a learning task is to understand the inherent patterns of a dataset and to learn to infer typically unobserved properties from descriptive features. Learning from data means that, based on a fully observed training set, a model can be trained to fulfill this task and to generalize to unseen data. The key ingredient to a successful generalization is that the training data shares the same distribution in terms of features and target property as the data the model will be applied to in the future. A bias violates this assumption and causes the generalization step to fail resulting in poor performance of the model.

The literature on *Transfer Learning* covers several kinds of distribution shift problems between observed and target data [25], whether it is due to a shift in the learning task, a shift in the data domain, or both. A special case of Transfer Learning is *Covariate Shift Correction* [26] where observed and target data share the same domain, e.g., the chemical compound space, and the same posterior distribution, but follow shifted data distributions. An example of covariate shift occurs in the drug discovery process [27] where predictive models are trained on known drugs but expected to generalize to unexplored compounds. If the models are expected to perform well on the observed and the target data, that is, if the target space contains the observed data, this special scenario is called a *Selection Bias* [28–30].

In all three problem formulations, the implicit or explicit assumption is that knowledge of the target domain is available, either in the shape of its distribution or a representative sample. The traditional and popular approach to solve this distribution mismatch is then to weigh the training compounds based on their estimated relevance in the target domain during the model training process [30–33].

However, if the target domain cannot be specified or is generally unknown, as it is in our problem statement, none of these approaches can be used.

*Active learning in chemistry*

Active Learning is a semi-supervised Machine Learning setting that utilizes information from a trained model to infer the samples which would most improve the model [10]. The main aim is to train models using fewer labels than would be required for random sampling as these are often expensive to obtain.

An Active Learning strategy consists of an initial model, usually trained on a small amount of randomly selected data; a query strategy, which is responsible for identifying the most informative samples; and a setting, which determines how those samples are obtained. A wide variety of query strategies have been proposed in prior work, but uncertainty-based strategies are the most common [10]. These strategies evaluate the confidence of the model on each sample, and samples with the lowest confidence (highest uncertainty) are considered the most informative. New samples can be obtained from an unlabelled pool (*pool-based*) or synthesized de novo (*query-synthesis*). In practice, pool-based Active Learning is typically preferred as synthesized samples are often difficult to label, or simply invalid [34].

In cheminformatics, Active Learning has demonstrated the potential to improve the quality of models while reducing the amount of data required [35]. For example, Smith et al. used Active Learning to train a model for molecular energetics that outperformed a model trained using random selection while using only $10\%$ of the available labels [35]. Active Learning has also been applied to the fields of drug-discovery [36], toxicity prediction [37], chemogenomics [38], and others [39].

In contrast to the approach presented in this paper, Active Learning attempts to select samples which *improve the current model*. The selected samples are not necessarily transferable to other models [40]. Additionally, Active Learning intentionally seeks to bias the dataset towards informative samples and does not aim to explore the space or improve the dataset quality.

*Bias in the chemical compound space*

Hert et al. [3] aim to quantify the bias of screening libraries towards biogenic molecules, given an estimate of the entire space and a specified optimal dataset, i.e., the optimal bias, by assessing the similarity between the observed and the optimal dataset. Given that the chemical space is estimated to contain at least $10^{60}$ molecules with 30 or fewer heavy atoms [41], stretching even today’s largest databases across that space to achieve the often idealized uniform distribution [5, 18] would result in very sparse coverage. The authors hence postulate that, as opposed to the aim to cover the entire space uniformly, biases toward specific domains are essential to enable the successful performance of models and researchers within those domains. In agreement with this, in this paper, rather than aiming to cover the entire compound space, we suggest a technique that mitigates the bias within an observed dataset while preserving its bias within the compound space. Therefore, despite improving the dataset quality, we preserve the dataset’s specialization to its domain.

Sieg, Flachsenberg, and Rarey [2] investigated multiple benchmark datasets for structure-based virtual screening such as DUD, DUD-E, and MUV, and discovered that they are all inherently biased since they have grown depending on human decisions based on individual assumptions and goals. When screening for specific properties, these biases persist and eventually find their way into models trained on these datasets resulting in a negatively impacted model performance [19]. Attempts to mitigate the dataset biases during screening evolve around different sampling techniques or strategic omission of features [2]. While those are feasible approaches in large databases, they mean a substantial loss of information in small datasets [42] such as those we are working with. Here, the long-term goal must be to smooth out the biases within the dataset domain and improve the data quality in the future.

**Fig. 3:**
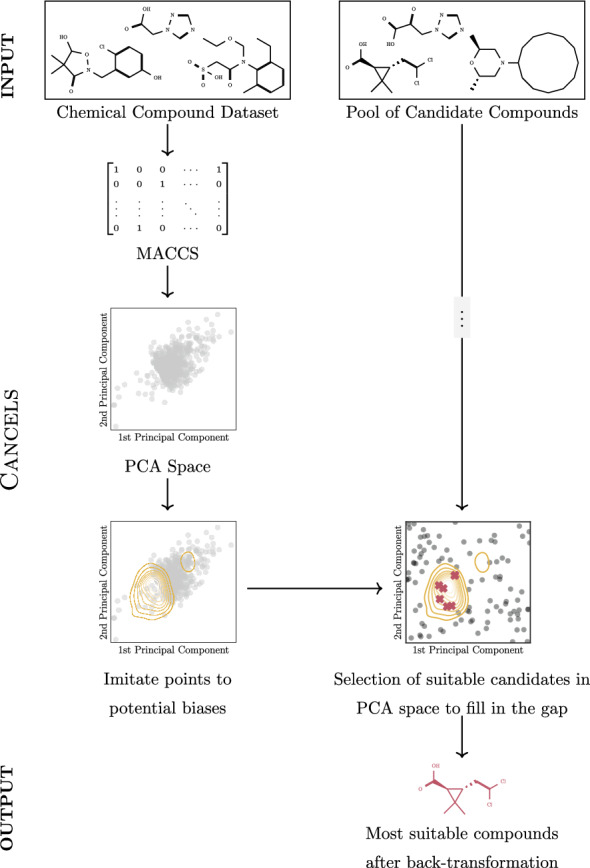
Overview over cancels

## Proposed method

When presented with a potentially biased dataset, we would like to identify present biases and mitigate them in subsequent experiments. The imitate and mimic algorithms presented in the previous section deal with this problem for real-valued, numeric, and tabular data, but are not applicable to the chemical compound space. Compounds can be represented in a variety of different ways, e.g., as SMILES, molecules, or MACCS fingerprints, but none of these representations fit imitate’s and mimic’s criteria. Additionally, to mitigate a bias, both algorithms generate data that smoothes out the distribution of the biased dataset. However, random generation of chemical compounds will most likely not result in meaningful and feasible compounds. We address both problems with our novel algorithm, cancels (**C**ounter**A**cti**N**g **C**ompound sp**E**cia**L**ization bia**S**).

The idea behind cancels is to represent the compounds in the potentially biased dataset as MACCS fingerprints because of their widespread use, fixed lengths, efficiency to compute, and solid performance in a diversity of applications [43]. Based on a comparison of different compound representations, we found that MACCS fingerprints also perform well in our case (see our experimental results and Fig. 11 for details). We then use *Principal Component Analysis (PCA)* to strongly reduce the dimensionality of the data and obtain Gaussian-like real-valued distributions as is necessary for imitate. In the PCA space, imitate can be applied, with adaptations (as discussed below), and point to potential biases. Data to mitigate the bias could be generated in this space, but not transformed back to the original space leaving the output hardly interpretable. Instead, we propose to use the PubChem [44] database as an unlabeled pool of candidates and project each of them into the PCA space. Rather than generating new data, cancels chooses from the candidates. As a result, we not only ensure that a back-transformation to the original compound space is possible, but also that the selected candidates to mitigate the bias are indeed feasible compounds. Figure 3 summarizes the procedure.

The remainder of this section discusses all involved steps in detail. It is organized in the order cancels uses it. Note that cancels draws from both imitate and mimic in the first three steps and when identifying compounds to mitigate the bias, respectively.

*Data transformation*

 Starting from a potentially biased set of compounds, we represent each of them using the MACCS fingerprint since it provides us with a fixed-length feature representation. MACCS fingerprints have been shown to include correlated features causing distance measurements to be flawed [45], however, we subsequently reduce the dataset dimensionality and thereby mitigate the effect of related features. At the same time, reducing the dimensionality overcomes the problem of binary features. cancels uses PCA to reduce the compound dataset expressed as MACCS fingerprints to the first $n_\text {PC}$ principal components. If $n_\text {PC}$ is sufficiently small (see Fig. 12 for a comparison of different values; we use $n_\text {PC}=5$ in our experiments), we can observe continuous non-discrete distributions over the axes to which imitate can be applied. Note that by using PCA, we implicitly operate in Euclidean space as opposed to the typical treatment of MACCS keys using Tanimoto distances. As pointed out by Martin and Cao [46], this decision can lead to more emphasis on the compounds’ length than their differences and future research should be dedicated to applying Multi-Dimensional Scaling (MDS) [47] to a Tanimoto distance matrix instead.

*Bias identification*

Once the compound dataset is transformed into PCA-space, imitate exploits the orthogonality of the principal components and analyzes the dataset distribution over each of them separately. Histograms or Kernel Density Estimators (KDE) evaluated over a grid approximate the data’s probability density. KDE is preferable for small datasets since it is less sensitive to the choice of grid whereas histograms are substantially faster to evaluate. Similar to imitate, we choose the type of density estimation based on the dataset size (with a threshold of 1000 compounds), and select the grid granularity that optimizes the corrected Akaike Information Criterion [48].

Using the density estimates on the grid as the targets and their square as weights, imitate fits a scaled and truncated Gaussian that models observed data as closely as possible but might over-estimate areas that are under-represented in the data. This discrepancy between observed data and fitted Gaussian points to potential biases. imitate’s weighted optimization (as explained in the "Background" section) is the key to this result: It puts more emphasis on higher density values during the optimization allowing room for error on lower densities under the premise that densely populated areas are more ‘trust-worthy’ than sparse ones. However, there is no guarantee that imitate identifies areas as biased that are actually populated in the compound space.

*Boundaries*

To alleviate the problem that imitate points to areas of the compound space that do not contain feasible compounds, we need to derive a method to provide the optimization process with boundaries. Luckily, the goal is to smooth out the distribution to obtain a Gaussian density. While this problem has only one global optimum, it has multiple local optima that bring equally smooth Gaussians at the cost of filling in more compounds. If imitate converges to a globally optimal solution that is outside the feasible compound space, we redirect it to the next best solution within the space unless the quality gap between the solutions is too extreme. The boundaries of the feasible compound space are extracted from the pool that is used to select bias-mitigating solutions.

In order to give the user control over the acceptable quality gap, we suggest a parameterized solution. Instead of using constrained optimization, we adjust the optimization target and weights. Out-of-bounds optimization targets are set to 0, and their weight is set to $w>0$ times the highest within-bounds weight (see the imitate paper [12] for details on the weights and optimization). A small *w* will have little impact on the optimization and the obtained Gaussian is not likely to change. The larger *w* is, the more strongly the optimization is forced to find a different solution. Intuitively, *w* quantifies the acceptable quality gap since errors on out-of-bounds targets can be translated to errors in high-accuracy regions with respect to the grid and the size of the out-of-bounds region.

**Fig. 4:**
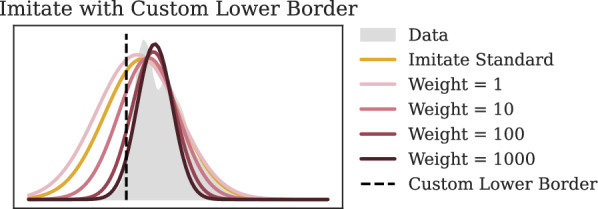
Comparison of different weights for imitate with a custom boundary

Based on a variety of preliminary experiments, we decided to use $w=10^3$ for our experiments since it is sufficiently strong to move the optimizer to a suitable within-bounds optimum unless there is no other reasonable solution. See Fig. 4 for a comparison of different choices for *w*. Once the Gaussian has been redirected, compounds need to be identified that are capable of filling in the gap.

*Identifying compounds to fill in the gap*

Univariate Gaussians fitted to each component separately can be combined into a multivariate Gaussian (see our previous work [13] for details) pointing to biases in PCA space. To mitigate these biases, compounds need to be identified that, when added to the dataset, smooth out its distribution by filling in the gap between present data and fitted Gaussian.

The mimic algorithm iteratively uses imitate to find flaws in initial clusters, scores, and adds points mitigating these flaws until it finds a bias-aware Gaussian clustering of the data. In each step, after obtaining a new target Gaussian from imitate, mimic scores all available points from other clusters and uses the scores to randomly select candidates to be added to the cluster. It stops once adding further points would not improve the fit of the Gaussian.

cancels adapts this procedure and exploits mimic’s scoring function to select compounds from the pool transformed into the same PCA space. Note that PCA as a dimensionality reduction technique is not invertible, hence we need to store the mapping of pool compounds from the original to the PCA space in order to infer knowledge from the chosen candidates. Given the target Gaussian from the previous steps, cancels scores each compound *c* in the pool with

$$\begin{aligned}s(c) = \mathbbm {1}_{g(c)d(c)\ne 0} \left( \log g(c) + n_\text {PC}\log d(c)\right) ,\end{aligned}$$

where *g*(*c*) is the density assigned by the Gaussian truncated at the triple standard deviation, *d*(*c*) measures the discrepancy between fitted Gaussian and available data at this point, and $\mathbbm {1}$ is the indicator function outputting 1 if the index condition holds true and 0 otherwise. After normalization, the calculated scores can be used as probabilities to randomly select compounds from the pool without replacement. cancels stops sampling compounds when adding further compounds would not improve the fitness of the Gaussian, that is when the likelihood of the Gaussian given the training set together with the additional data does not increase or the pool is exhausted.

Finally, cancels uses the stored mapping to obtain the original representation of the selected compounds. These compounds can be interpreted as suggestions of which experiments to carry out next, but since they have been selected randomly based on the calculated probability distribution, a direct interpretation might not be optimal. However, the selected compounds describe underrepresented areas. Analyzing their characteristics can help the researcher gain insights into which kinds of experiments fell short in the past, and manual selection of experiments that fill in this gap can be a valuable compromise between improved data quality and meaningful experiments with interesting results.

If the pool of candidate compounds is rather small, alternatively, a researcher might prefer to use the normalized scores for the entire pool directly and, rather than sampling from it, choose manually subject to additional criteria such as availability, price, or other properties not represented by the fingerprint. Note that adding only the compounds with the highest scores does not necessarily smooth out the dataset’s distribution but has the potential to create a new bias. Instead, the researcher would need to choose a large amount of highly-scoring compounds, some medium-score compounds, and even a few compounds with low scores. To simplify this process, we suggest repeatedly choosing a few compounds with high scores, adding them to the dataset, retraining cancels, and scoring the remaining pool until a desired number of compounds have been identified.

## Experimental setup

To showcase what cancels can reveal about a dataset and what insights can be won, we apply it to multiple datasets and analyze its results. Our use-case for this paper is biodegradability, however, cancels could also be applied to other domains such as drug development. Although cancels makes suggestions as to which compounds might be interesting to obtain labels for, analyzing these recommended compounds and their characteristics grants us more than that: It teaches us about weaknesses of the dataset and underrepresented areas that might cause a lowered model reliability regardless of the trained model. To quantitatively evaluate cancels’s performance though, we need to train a model to evaluate changes in accuracy. Note that no matter what we evaluate, cancels is in any case provided with only the MACCS fingerprints of the datasets, and has no access to labels or further data characteristics. In this section, we introduce our general experimental setup. We might deviate from this setup in single experiments depending on the question we aim to answer. All deviations are listed in the following section for the sake of reproducibility. Unless stated otherwise, we use the setup introduced here. Our implementation together with all experiments, results, and plots is publicly available on GitHub [49] for the sake of the reproducibility of results and to support further research.

*Datasets*

The main datasets we analyze in this paper are the EAWAG-SOIL [50] (short: SOIL) and EAWAG-BBD (short: BBD) datasets extracted from the enviPath platform [51–54]. Both datasets contain biodegradation pathways capturing the chemical changes of a given starting compound (we refer to this as a “root compound”) during biotransformation. SOIL and BBD contain 343 and 248 root compounds, respectively. We prepare both datasets by extracting the compounds’ MACCS fingerprints, and, to investigate the dataset development over time, join the year of publication of each pathway to its root compound where possible (299/343 root compounds in SOIL have years, and 215/248 in BBD) as well as use categories from the PubChem database [44].

For a large-scale experiment demonstrating how the application of cancels can help improve the classification accuracy, SOIL and BBD are too small to yield statistically reliable indications. Instead, in this case, we use the substantially larger Tox21 dataset [55, 56] containing 11093 compounds and similarly obtain MACCS keys as input features as pre-processed by Stepišnik et al. [43].

To put SOIL and BBD and their development over the years in a frame of reference, we downloaded all unique SMILES from the PubChem database to obtain an estimate for the span and the density of the compound space.

As pools for cancels to select compounds from, we use the subset of PubChem with an “Agrochemical” flag to be able to extract the same use categories we obtained for SOIL and BBD. When experimenting with Tox21, we split it into subsets so no external pool is necessary (see the following section for details).

*Classifiers, evaluation, and stability*

Tox21 is a dataset with multiple labels, hence we use a multi-label classifier to predict its labels. To achieve the most stable performance among runs and reduce the effect of randomness induced by the classifiers, we train Ensembles of Classifier Chains (ECCs) [57] with 10 chains per ensemble. We evaluate the classifier performance using *Multilabel-Accuracy* (short: Accuracy)

$$\begin{aligned} \text {acc} = \frac{\#\text {TP} + \#\text {TN}}{\#\text {TP} + \#\text {TN} + \#\text {FP} + \#\text {FN}} \ . \end{aligned}$$

Here, $\#\text {TP}$ and $\#\text {TN}$ count the number of correctly predicted positive and negative labels, respectively. Similarly, $\#\text {FP}$ and $\#\text {FN}$ count the number of mispredicted labels.

To achieve statistical stability and ensure the significance of observed patterns, we repeat every experiment 100 times under different dataset splits and report the average results together with $95\%$ confidence intervals.

## Results and discussion

cancels is a method that, given only an unlabeled dataset, searches for biases and underrepresented regions and suggests additional compounds that can improve the dataset quality. As such, we will use cancels as a tool to identify flaws in the dataset and investigate if the suggested compounds can indeed help improve the performance of subsequently trained models. This section investigates several questions ranging from if the bias spiral discussed in the introduction can indeed be observed in the datasets to what can be won by using cancels. Unless specified explicitly, all experiments have been set up as outlined in the "Experimental setup" section.

**Fig. 5:**
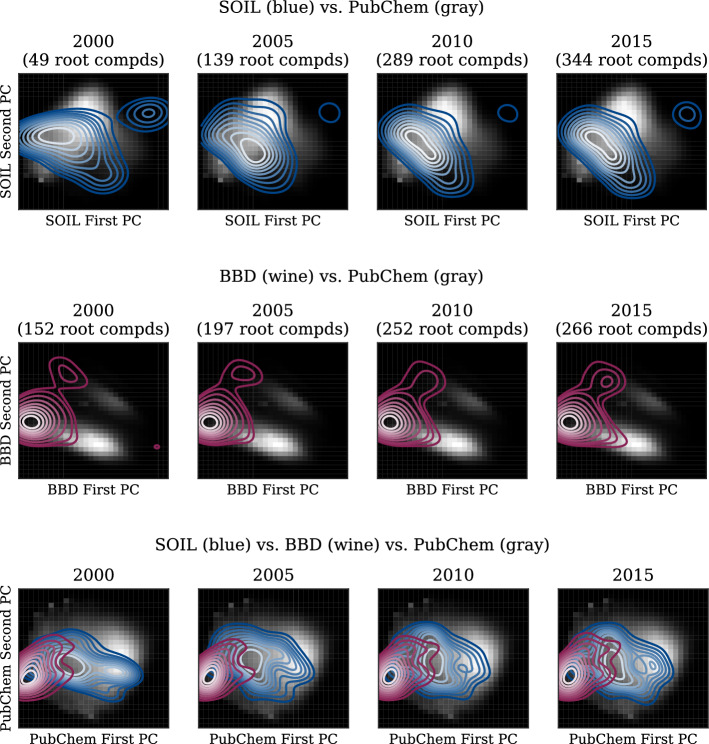
Qualitative dataset development for SOIL and BBD root compounds in relation to the compound space represented by the PubChem dataset and visualized in the PCA spaces obtained from SOIL (top), BBD (center), and PubChem (bottom). In all three datasets, white represents the highest density

**Fig. 6:**
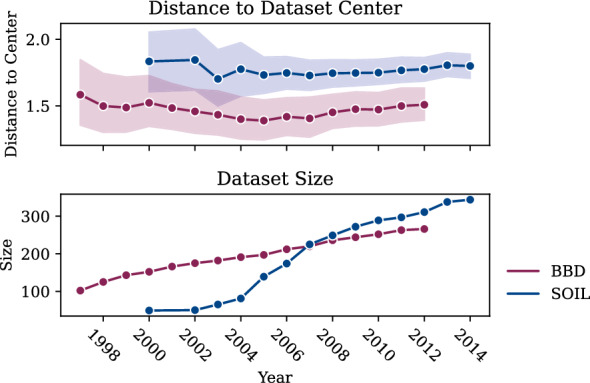
Quantitative development of SOIL and BBD root compounds in terms of the compound’s average distance to their center (top) and their dataset size (bottom)

*How did the datasets develop over time?*

Independent of if a model is in place to support the choice of which experiments are the most promising or not, we can make the most reliable assumptions on the outcome of experiments for compounds that are similar to those we observed before. We hypothesize that this reliability shapes the process of further experimentation and hence induces specialization to the part of the compound space that is already well populated while exploration of other parts of the compound space falls short.

This hypothesis seems to be confirmed for the development of the SOIL and BBD datasets. Figure 5 illustrates the development of the root compound datasets from the year 2000 to 2015. We use the PubChem database as a lower boundary for the space of feasible compounds (i.e., PubChem measures the already discovered compound space). The true space is even larger but has not yet been fully explored [14]. Regardless, neither SOIL nor BBD covers the entire space—the datasets are specialized to their respective domains. Both datasets consist of one main group of compounds and a second group that is structurally different from the first one. In SOIL, this smaller group mainly corresponds to sulfonamides typically acting as antibacterial and antifungal agents. In BBD, it corresponds to compounds containing groups of multiply oxidated elements such as sulfates and nitro compounds. We can observe that, although compounds are continuously being added to the datasets, their distributions seem stationary and the gaps between the main and the small groups are never closed.

Figure 6 further quantifies this suspicion. For both datasets, during the first years, the average distance of compounds to the center decreases indicating that compounds were added close to the center in the already populated areas. In later years, the average distance to the center has a slight upward trend, however, the standard deviation is decreasing at the same time indicating a shift of the center to another already populated area. In both cases, no new areas of the compound space are being explored although new compounds are continuously being added. Additionally, a small standard deviation implies a small area a model specializes to while other more sparsely populated areas are less reliably predictable.

**Fig. 7:**
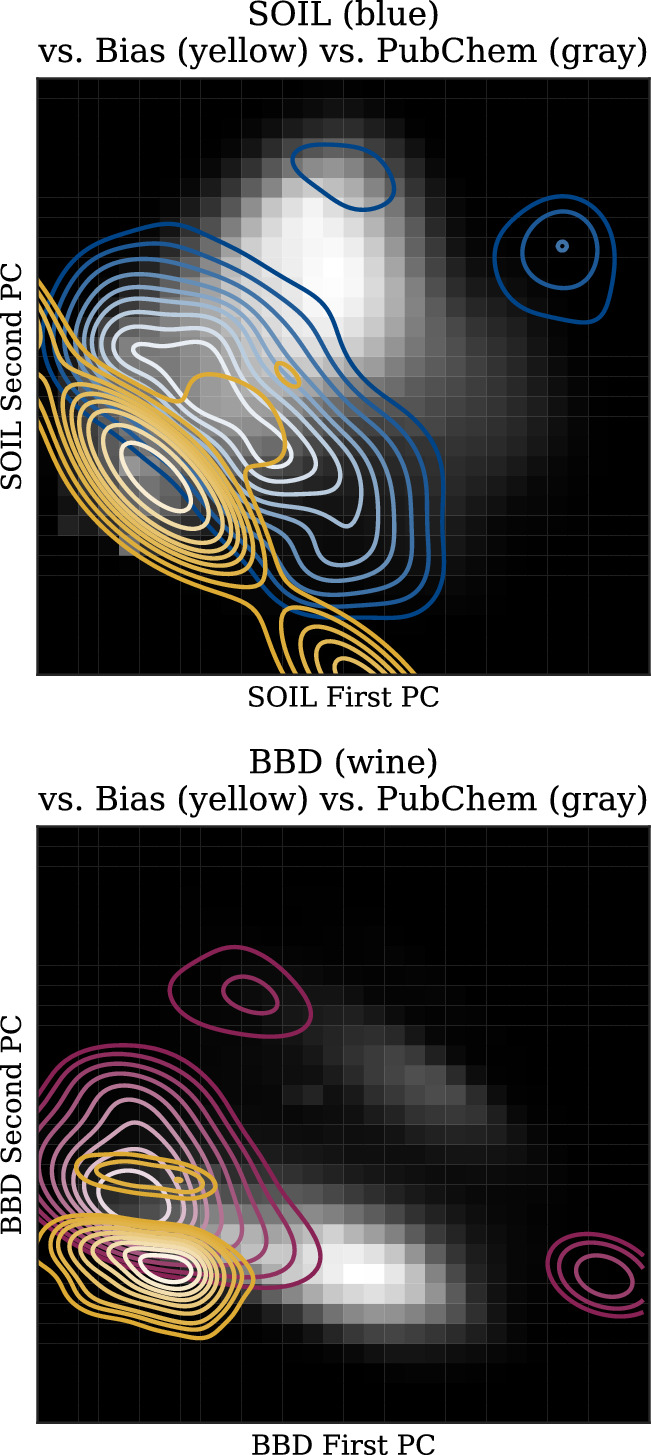
Potential biases detected by cancels for SOIL (top) and BBD (bottom) visualized in their respective PCA spaces against the PubChem compound space

*Which underrepresented regions can* cancels *detect?*

Application of cancels to the SOIL and BBD datasets reveals the underrepresented regions displayed in yellow in Fig. 7. When comparing the datasets and those regions to the entire compound space estimated using PubChem, we can see that mitigating these biases, while potentially improving the dataset quality, does not generalize towards covering the entire chemical space but rather smooths out the dataset’s distribution locally while retaining the specialization to the dataset’s domain.

One interesting observation is that cancels suggests adding compounds on the outer ranges of PubChem rather than its center. Sampling new compounds randomly would result in a distribution shift towards that of PubChem and the dataset would lose its focus on the domain for which it is designed.

Note that the indicated areas focus on regions within the compound space due to the boundaries introduced in the "Proposed method" section, so finding suitable compounds that mitigate this bias is possible.

**Fig. 8:**
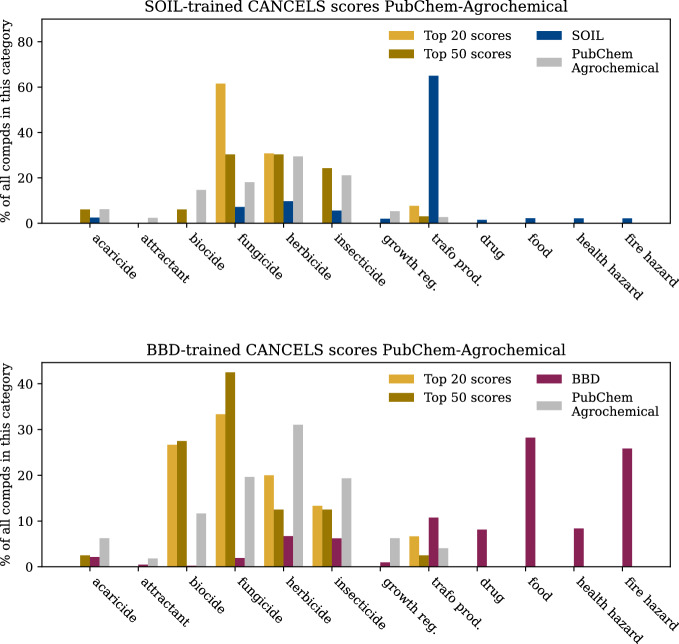
Qualitative evaluation of the top 20 and top 50 compounds suggested by cancels to mitigate the detected biases in SOIL (top) and BBD (bottom) in comparison to the respective dataset’s compounds and the “Agrochemical” subset of PubChem. Note that categories are non-exclusive

*Which kinds of compounds does* cancels *suggest to mitigate the bias?*

To fill in the underrepresented regions identified in the previous experiment, we offer cancels a pool of compounds to choose from. This pool is assembled from those compounds in the PubChem database that carry an “Agrochemical” flag. The reduction to this subset was necessary to enable us to extract the same auxiliary information from the pool data that is already available for the SOIL and BBD datasets. Figure 8 displays the frequency of relevant, non-exclusive labels for the entire pool (in gray) as well as the input dataset (SOIL in blue, BBD in wine) and the top 20 and top 50 candidate compounds to mitigate the bias.

We observe a shift towards fungicides and herbicides for SOIL and biocides and fungicides for BBD in the recommendations for both datasets. This is a meaningful result since both categories are under-represented in the datasets by design, but seem relevant to add as they are structurally similar in order to train models on the datasets. Comparison with the entire pool shows that cancels specifically targets compounds belonging to these categories—they do not reflect a general trend of the pool. Note that these results have been obtained although cancels was never presented with these categories but only the MACCS representations of compounds.

**Fig. 9:**
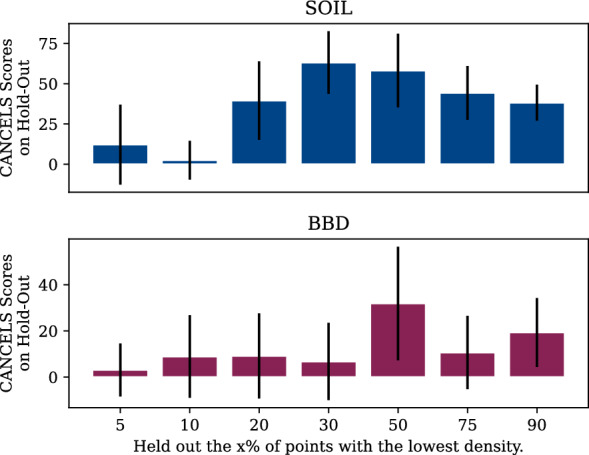
While holding out $x\%$ of the SOIL (top) and BBD (bottom) datasets, we train cancels on the rest. Bar heights represent average scores of the holdout set with their corresponding uncertainty intervals (black lines)

*Cross-check: does* cancels *perform as expected?*

To cross-check that cancels is working as intended, we carry out an additional experiment. Training a kernel density estimator to model the dataset’s density, we sort all compounds by their assigned densities. Holding out the $x\%$ of the dataset with the lowest density, we use cancels on the rest and score the held out compounds. Intuitively, removing data from a dataset should reduce its quality and result in high scores for the removed data aiming to retrieve the original dataset quality.

The results are shown in Fig. 9. We see that for low percentages *x*, the scores are generally low. This is expected since outliers will be removed first and cannot be expected to score highly. For high *x* the average scores are decreasing again. This is also expected since cancels is applied to a very small portion of the dataset only and, by design, makes conservative estimates resulting in high scores only for some of the removed compounds. The peak is at $x=50\%$ where both effects are minimal. Overall, cancels’s general behavior fits our expectations.

We notice a few irregularities in the patterns deviating from a smooth ascend to and descend from the $x=50\%$ peak. These irregularities stem from a change in the underrepresented area cancels points to and are an indication of a bias in the dataset: If the dataset was smooth and unbiased, removing those $x\%$ of compounds with the lowest density would narrow the dataset to its center (or, if there are multiple clusters, to their centers) equally from all sides. In this case, the estimated Gaussian would stay consistent over all $x \le 50\%$ and potentially even for higher ones. Hence, since we observed jumps, we can conclude that a bias must be present even from this perspective.

**Fig. 10:**
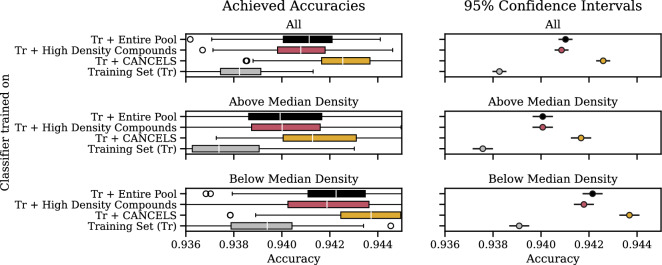
Dividing the Tox21 dataset into a training set, a pool, and a test set, we train a classifier on either the training set only, the training set together with the entire pool, the training set plus cancels-based compound selection, and the training set plus a selection that feeds the biases instead of mitigating it. The box plot (left) displays the results in terms of accuracy when evaluating the trained models on the test set. A confidence interval plot (right) indicates that compound selection using cancels is significantly better than all other options

*Can* cancels *improve the model performance?*

To assess the relevance of the compounds suggested by cancels, we use the Tox21 dataset (see our experimental setup) due to its size and set up an experiment as follows: In each of 100 runs, we randomly hold out $40\%$ of the dataset as a test set (4437 compounds), offer $40\%$ of the remaining data as a pool (4437 compounds), and use the rest for training (2219 compounds). Due to the sampling of the relatively small training set, a statistically small bias can be introduced whose effect is smoothed out by the 100 runs. Note that we do not introduce an artificial bias into the dataset with our sampling procedure but instead retain the original bias we suspect to be in the dataset.

Based on the training set, we select additional compounds from the pool in four different scenarios: We can select (i) no additional compounds, (ii) $n_{\textsc {Cancels}}$ compounds suggested by cancels, (iii) $n_{\textsc {Cancels}}$ compounds that feed rather than mitigate the bias based on density-based random sampling (i.e., we sample based on the dataset distribution directly), or (iv) all available additional compounds (i.e., the entire pool).

A classifier is then trained on the training set together with each selection of additional compounds and evaluated on the test set.

Figure 10 shows that compound selection using cancels not only is better than continuing to feed the bias but also than using the entire pool! A repeated measures ANOVA with posthoc Tukey HSD test [58, 59] confirms that these results are statistically significant under significance level $\alpha =0.01$.

Splitting the test dataset along the compounds’ median density reveals that this effect is particularly strong in the low-density areas. This is an essential result since it supports the exploration of the space that breaks the bias spiral and has the potential to lead to global rather than local optimization.

**Fig. 11:**
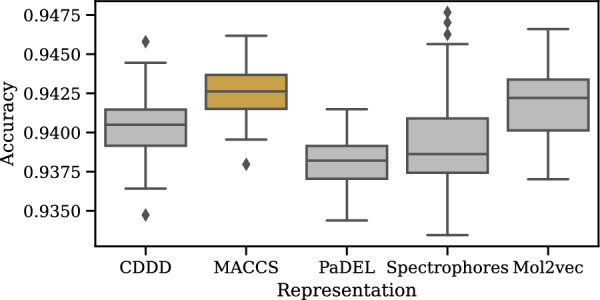
Influence of different compound representations on cancels’s performance

*How does the compound representation affect the performance?*

Using a MACCS fingerprint as a compound’s feature representation for training a model is widely popular [43] due to the computational speed and the solid performance in different applications. However, cancels’s compound feature representation is independent of that used by the model. To investigate which representation performs best in cancels, we repeat the previous experiment with the following competitors to MACCS fingerprints: (i) *Continuous Data-Driven Descriptors (CDDD)* [60] obtained from an RNN autoencoder, (ii) *PaDEL* [61], a set of 1875 2D and 3D molecular properties, (iii) *Spectrophores* [62] calculated from 3D properties of molecules using affinity cages, and (iv) *Mol2vec* [63], a neural network-based embedding similar to the word2vec models used in Natural Language Processing trained to embed structures co-appearing frequently near each other in latent space. For all competitors, we obtained the pre-processed datasets from Stepišnik et al. [43].

Figure 11 illustrates the results: The differences between representations are small. MACCS and Mol2vec perform slightly better than the rest, and MACCS fingerprints additionally show a smaller variance among runs. Ultimately, the right choice of feature representation depends on the application and should be investigated individually, but in our use case, using MACCS fingerprints for cancels seems well justified.

**Fig. 12:**
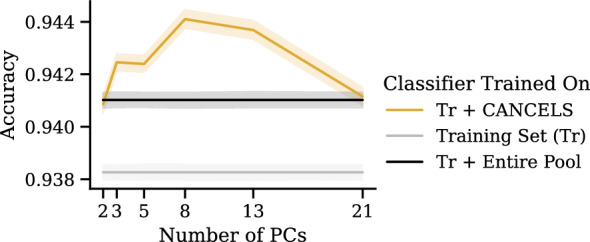
Influence of the number of principal components used in cancels’ dimensionality reduction

*How does the number of principal components influence the performance?*

Choosing the correct number of principal components for PCA in cancels in an unsupervised setting is a difficult task since we have no feedback as to which number performs best. Intuitively (and following the Central Limit Theorem), the smaller the number $n_\text {PC}$ of principal components, the more closely our dataset distribution will resemble a Gaussian. At the same time, the higher $n_\text {PC}$, the more variance in the dataset we can explain using the components. That is, a dataset can be modeled perfectly if its dimensionality matches $n_\text {PC}$, but information will be lost if the dimensionality is reduced. We can see both aspects in Fig. 12 where there is a peak around $n_\text {PC}=8$ indicating that the results presented here (with $n_\text {PC} = 5$) could have been better, but our estimated value is reasonable. To choose a suitable value for $n_\text {PC}$, as a rule of thumb, we suggest trialing different values and visualizing the dataset distribution over the resulting components. A solid choice is the largest value that shows Gaussian-like distributions over all components. In future research, we will investigate how to choose $n_\text {PC}$ automatically.

**Fig. 13:**
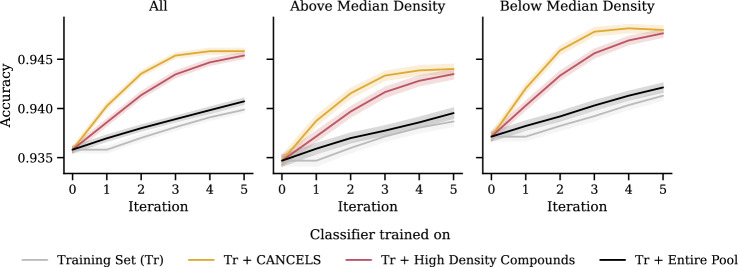
Iterative application of cancels and all competing baselines (see Fig. 10) on the Tox21 dataset: In each of the five iterations, the compound selection takes place based on the training set and the selected compounds from previous iterations. For cancels, the accuracy improves upon all other selection strategies

**Fig. 14:**
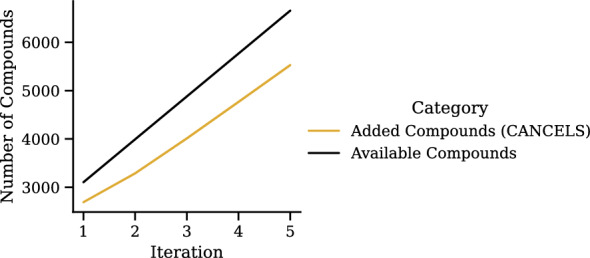
Number of added compounds in an iterative application of cancels

*Can iterative application of* cancels *improve the accuracy even further?*

The previous experiments showed an improvement in accuracy for cancels-based compound selection, especially in lower-density areas of the data space. To investigate the long-term effect, we carry out a similar but iterative experiment where we randomly split the pool into 5 equally-sized sub-pools. In each of five iterations, we select additional compounds from the corresponding sub-pool based on the training set and the selections from all previous iterations. As before, we select the same number of points for both cancels-based sampling and sampling based on the data density in every iteration to ensure a fair comparison. Note that an iterative application of cancels can help obtain a smoother result since the selection of suitable additional compounds is a randomized process. Particularly when working with multiple dimensions, selecting one compound that fills in a gap in one dimension can create artifacts in others that need to be smoothed in subsequent iterations. If the data is sufficiently Gaussian, however, no further compounds are added. More restrictive definitions of what is ‘sufficient’ can be implemented before each round of compound selections, for example using a statistical normality test such as the Shapiro-Wilk test.

Figures 13 and 14 summarize the impact of cancels on each of the iterations. Firstly, we observe that three iterations seem sufficient to smooth out the dataset distribution. Additional iterations have no effect and the accuracy is saturated. After three iterations, cancels has selected only about 4000 compounds and still largely outperforms the entire pool with about 7000 compounds. The red line (“Tr + High Density Compounds”) stands for training on the training set together with a random sample from the pool. Since the pool follows the same distribution as the dataset, sampling from it will result in mostly compounds in dense areas, but few compounds from sparse areas can also find their way in, so the red line eventually catches up with cancels. This effect is an anomaly due to our experimental design and will no longer be observed if the pool’s distribution does not match that of the dataset and the test set. In summary, selecting the right compounds not only improves the data quality but also is substantially more economical as it means carrying out fewer experiments.

In practice, improving the dataset quality is not the only goal—a researcher also aims to make decisions regarding their data collection based on their current interests, projects, and goals. To achieve a healthy balance, we suggest one or two iterations of cancels after each interest-driven addition to the dataset before the dataset is fit for its upcoming tasks.

## Conclusion

Predictive modeling can support the development process of new chemicals, however, those models specialize to the data provided, and solid performance can only be guaranteed in densely populated areas of the compound space. Avoiding carrying out experiments with a very uncertain result, new additions to the dataset will most likely stem from already densely populated areas where the prediction reliability is high. Over the years, this results in a stronger over-population of already over-populated areas and a shrinking applicability domain of trained models inducing a specialization bias.

To break this spiraling specialization cycle, in this paper, we propose cancels, a novel technique to investigate a dataset independently from a specific model, create awareness of underrepresented areas, and suggest additional compounds that can help mitigate the bias. So far, cancels is unique in many regards: (i) It generally improves the dataset quality in a model-independent fashion while other methods are only designed to support the training process of one specific model, (ii) while generalizing the dataset and enabling further targeted exploration of the compound space, cancels does not lose the desired specialization to a certain domain when suggesting additional compounds, and (iii) cancels’s outputs are interpretable and can be used to investigate different aspects of a dataset as demonstrated in our extensive set of experiments.

Our various experiments indicate that on two real-world datasets, SOIL and BBD, a continuous specialization can indeed be observed which renders these datasets a valid use-case for cancels. Interpretation of the results suggests that a focus on fungicides and herbicides or biocides and fungicides for SOIL and BBD, respectively, would increase a trained model’s applicability domain and hence improve its performance. Validation of cancels on the Tox21 dataset shows that careful selection of future experiments can not only reduce the total amount of experiments to be carried out but also improve the performance of predictive models by a significant margin.

All results presented in this paper have been obtained based solely on the compounds’ MACCS keys. Future research will investigate how auxiliary information can be integrated in an effective way where available. Additionally, we aim to make cancels fully automated for the simplest usage possible. As such, we aim to automatically infer parameters such as the number of principal components from the dataset and context, for example using information criteria that incorporate a measure of Gausseanity but penalize for every dimension lost. Overall, we hope that cancels can be of use to help researchers understand the datasets they are dealing with and to improve their quality early on to improve their usability universally.

## Data Availability

The SOIL [64] and BBD [65] datasets are publicly available in the enviPath platform [66]. PubChem [67] and the Tox21 [68] dataset are also publicly available. All pre-processed datasets supporting the conclusions of this article are included within the article and its additional files. For Fig. 11, we used preprocessed datasets from Stepišnik et al. [43] and obtained the authors’ permission to make these sets available with our implementation. We provide all implementations, scripts for experiments, and experimental results on GitHub [49] to enable the reproducibility of our results. Additionally, cancels has been integrated into the PyPI package imitatebias [69], where we provide comprehensive documentation of the user interface.

## Abbreviations

BBD: EAWAG-BBD dataset
CADD: Computer-Aided Drug Design
CANCELS: **C**ounter**A**cti**N**g **C**ompound sp**E**cia**L**ization bia**S**
CDDD: Continuous Data-Driven Descriptors
ECC: Ensemble of Classifier Chains
ICA: Independent Component Analysis
i.i.d.: Independent and identically distributed
KDE: Kernel Density Estimation
MACCS: Molecular ACCess System
PCA: Principal Component Analysis
SMILES: Simplified Molecular-Input Line-Entry System
SOIL: EAWAG-SOIL dataset

## Footnotes

1 The Central Limit Theorem states that a sequence of independent and identically distributed (i.i.d.) random variables converges almost surely (that is, the probability of the convergence is 1) to a Gaussian [23]. Since we can typically assume that real-world measurements are not perfectly i.i.d. but rather combinations of different effects, we often observe this effect.
